# What is the importance of vaccine hesitancy in the drop of vaccination coverage in Brazil?

**DOI:** 10.11606/S1518-8787.2018052001199

**Published:** 2018-11-27

**Authors:** Ana Paula Sayuri Sato

**Affiliations:** 1 Universidade de São Paulo. Faculdade de Saúde Pública. Departamento de Epidemiologia. São Paulo, SP, Brasil

**Keywords:** Immunization Programs, Vaccination Coverage, Vaccination Refusal, Anti-Vaccination Movement, Programas de Imunização, Cobertura Vacinal, Recusa de Vacinação, Movimento contra Vacinação

## Abstract

The successful *Programa Nacional de Imunizações do Brasil* (Brazilian National Immunization Program) has been experiencing a major challenge with regard to vaccination coverage for children, which has been dropping. Several aspects are related, but certainly vaccine hesitancy has been strengthening itself as one of the main concerns of Brazilian public administrators and researchers. Vaccine hesitancy is the delay in acceptance or refusal despite having the recommended vaccines available in health services, being a phenomenon that varies over time, over location and over types of vaccines. Hesitant individuals are between the two poles of total acceptance and refusal of vaccination. Vaccine hesitancy is nothing new in European and North-American countries, and even in Brazil, it has been studied even if under another name. The drop of vaccination coverage observed from 2016 on reiterates the relevance of the theme, which must be better understood through scientific research.

## RESUMO

O exitoso Programa Nacional de Imunizações do Brasil tem vivenciado grande desafio com relação às coberturas vacinais infantis, que têm apresentado queda. Diversos aspectos estão relacionados, mas certamente a hesitação vacinal vem se fortalecendo como uma das principais preocupações dos gestores e pesquisadores brasileiros. Hesitação vacinal é o atraso em aceitar ou a recusa das vacinas recomendadas quando elas estão disponíveis nos serviços de saúde, sendo um fenômeno que varia ao longo do tempo, do local e dos tipos de vacinas. Indivíduos hesitantes situam-se entre os dois polos de aceitação e recusa total da vacinação. A hesitação vacinal não é novidade em países europeus e norte-americanos e, mesmo no Brasil, ela já vem sendo estudada ainda que sob outra denominação. A queda das coberturas vacinais observadas a partir de 2016 reitera a relevância do tema, que deve ser mais bem compreendido por meio de investigações científicas.

## INTRODUCTION

Eradication of smallpox and control of vaccine-preventable diseases were possible throughout the world^1^ ^–^ ^3^ because of vaccination, through successful immunization programs. Global initiatives have contributed substantially to the development of these programs in middle- and low-income countries. The Expanded Programme on Immunization (EPI) of the World Health Organization (WHO), established in 1974, promoted access to vaccination, indicated by the increase in coverage of the diphtheria-tetanus-pertussis (DTP) vaccine^4^. The challenge became the equity of access to new vaccines. Thus, the Global Alliance for Vaccines and Immunization was created in 2000 with the objective of providing this access to every social stratum in low-income countries^1^.

The *Programa Nacional de Imunizações do Brasil* (Brazilian National Immunization Program – PNI) is recognized for promoting the free vaccination of more than 15 antigens. It has been getting increasingly complex, both by expanding the number of vaccines provided as by the diversification of immunization schedule^5^. Paradoxically, such advancement brings challenges inherent to its evolution, because the disease control from high vaccination coverage influences the perception of risks and benefits for vaccination^6^.

Since the 1990s, the vaccination coverage for children were above 95%^5^, which indicates good adherence of the population to vaccination. However, since 2016 such coverage has been declining about 10 to 20 percentage points^a^. This was unexpected and came with an increase in infant and maternal mortality^b^. Measles epidemics in Roraima and in Amazonas are immediate consequences of the decrease in vaccination coverage^c^.

Several factors are related to this drop, whether the weakening of the Brazilian Unified Health System (SUS), or of the technical aspects, such as the development of the new immunization information system, whether the social and cultural aspects that affect the acceptance of vaccination^5^ ^,^ ^7^ ^–^ ^10^.

Anti-vaccine movements are growing and being strengthened by the increase in incorrect health information shared especially in the internet^11^. Vaccine hesitancy is an old concern of researchers from European and North American countries^12^ ^–^ ^14^. In Brazil, few studies were developed about this phenomena^10^, but vaccine hesitancy has become increasingly evident.

The drop in vaccination coverage for children and its visible consequences justify the effort to better understand vaccine hesitancy in Brazil. Thus, the objective of this comment is to present the definition and factors related to vaccine hesitancy, as well as discuss its importance in the Brazilian context.

### Vaccine Hesitancy: Definition and Related Factors

Hesitancy comes from the Latin word *hæsitātĭō* and is defined by the state of hesitating, i.e. of being indecisive at the moment of making decisions^15^. In 2012, the WHO formed the Strategic Advisory Group of Experts – Working Group (SAGE-WG) to define vaccine hesitancy, to understand its magnitude as well as the factors that influence it, and to systematically gather evidence of interventions in public health^16^.

Vaccine hesitancy is defined as the delay in acceptance or refusal of recommended vaccines, despite their availability in health services^16^. This behavioral phenomenon is quite complex compared to its determinants (which involve cultural, social, and economic aspects), and varies over time, over location and over types of vaccines^12^ ^,^ ^13^ ^,^ ^16^.

It should be understood as a continuum between those who undoubtedly accept and those who undoubtedly refuse all vaccines, i.e. hesitant individuals are between these two extremes. They form heterogeneous groups, in which a few people only accept some vaccines and others delay it on purpose, not accepting the recommended vaccination schedule. In smaller proportion, there are those who refuse only some vaccines and those who still have doubts about the decision of having a vaccination or not^13^ ^,^ ^16^ ^,^ ^17^.

This behavior is influenced by several interrelated factors such as confidence, complacency and convenience, known as the “3Cs” model, proposed by WHO in 2011. Confidence is about the effectiveness and safety of vaccines, the health system that delivers them, and the public administrators' motivations for recommending them. Complacency results from the low risk perception of contracting diseases so that vaccination would be considered unnecessary. Finally, convenience considers physical availability, willingness to pay, geographical accessibility, ability to understand and access to health information^16^.

In 2012, the SAGE-WG defined a matrix of determinants to vaccine hesitancy considering individual and contextual characteristics and specific vaccination issues. In short, the domain of contextual influences includes historical, geographical, political, socioeconomic, cultural, religious, and gender aspects, as well as communication and media, influence of leaders and perception over the pharmaceutical industry. Individual influences are prior experiences with vaccination, beliefs and attitudes toward health, confidence to the health system, link with health professionals, vaccine risk perception, and vision of immunization as a social norm against that in which vaccination is not required or is harmful. Finally, the specific aspects of vaccine include risks and benefits, vaccination schedule, method of administration, introduction of a new vaccine or formulation, costs and supply of vaccines^13^ ^,^ ^16^.

We emphasize that vaccine hesitancy and its determinants vary over time and are specific to each context. Systematic reviews reiterate this heterogeneity, indicating the need of strengthening this knowledge in different contexts and qualifying immunization programs to approach it^12^ ^,^ ^13^ ^,^ ^18^. A study with public administrators of 13 countries showed that most of them interpreted hesitancy as vaccine refusal and some considered it a small problem^12^. Factors also differed among countries^12^; for example, the caregiver's high educational level and favorable socioeconomic status do not influence vaccine hesitancy always in the same direction^16^.

A study with 67 countries, including Brazil, found that the general feeling in relation to vaccination is positive, but with a great variability. Safety outstood as the aspect that brings negative feelings, particularly in Europe. In addition, countries with high educational levels and good access to health services had the lowest rates of positive feelings about vaccination^18^. Percentages of Brazilians who responded to disagree with the importance, safety, and effectiveness of vaccines were 0.7%, 6.1%, and 4.5%, respectively, very below other localities^18^.

Despite vaccine hesitancy being a recognized problem, its measurement is still a challenge. The SAGE-WG also developed, from other previously validated questionnaires^19^ ^,^ ^20^, three instruments about vaccine hesitancy with different types of questions: basic closed-ended, Likert scale and open-ended^21^. The Box presents the instrument in Likert-scale translated into Portuguese, but still not validated.

**Box:** Instrument to study the hesitancy to child vaccination applied to parents or caregivers in Likert scale of 5 points.

How much do you agree with the following statements about vaccines? Please indicate your answer using the scale below:
1 = strongly disagree; 2 = disagree; 3 = neither agree nor disagree; 4 = agree; 5 = strongly agree
L1. Vaccines are important for my child's health.
L2. Vaccines are effective.
L3. Having my child vaccinated is important for the health of others in my community.
L4. All childhood vaccines offered by the government are beneficial.
L5. New vaccines carry more risks than older vaccines.
L6. I trust the information I receive about vaccines from the immunization program.
L7. Getting vaccines is a good way to protect my child from disease.
L8. Generally I do what my health care provider recommends about vaccines for my child.
L9. I am concerned about serious adverse effects of vaccines.
L10. My child does not need vaccines for diseases that are not common anymore.

Source: Adapted from Opel et al.^19^ and Larson et al.^21^

And how to deal with the vaccine hesitancy? Systematic reviews concluded that there is no strong evidence to recommend specific interventions. In general, most interventions had more than one component and those directed to the increase in knowledge (communication strategies, media, social mobilization, information tools for health professionals) outstood. Also, we can cite interventions based on non-financial incentives and on scheduling strategies, as well as call-up for vaccination directed to the target population^22^.

### The Anti-Vaccine Movement and its Consequences

The anti-vaccine movement is as old as the vaccination itself. In the United Kingdom, there were caricatures of smallpox vaccine since the 1800s. The compulsory vaccination caused resistance of individuals that considered it an invasion of freedom over their own body. In the United States, court fights against compulsory vaccination were not rare in the 1920s. Nevertheless, in high-income countries, the 1950s and 1960s were considered the “golden age of vaccine acceptance” with the introduction of universal vaccination against poliomyelitis and measles-mumps-rubella (MMR), resulting in the significant decline of these diseases. On the other hand, middle- and low-income countries started this phase in the 1970s, with the support of EPI^11^.

From 1980 on, the controversy of whole-cell pertussis vaccine started, culminating in the development of acellular vaccine. In the United States, a passive surveillance system was created for adverse events following immunization, for, besides better monitoring these events, providing more transparency to the population. In the 1990s, articles about the possible association between MMR vaccine and Crohn disease were published and later, about the connection of this vaccine with autism. Even after publication of other studies refuting this relationship, this feeling still lingers in the population and such information is disseminated nowadays in a more agile manner through the internet^11^.

The consequences are the frequent epidemics of vaccine-preventable diseases, as measles and whooping cough, that currently occur throughout the world^23^, and the threat of reintroduction of poliomyelitis in regions where it had been already eliminated^24^. In Europe, in the first eight months of 2018, more than 41,000 cases of measles occurred^d^. In the United States, a substantial proportion of measles cases occurred in intentionally-unvaccinated individuals. Similarly, vaccine hesitancy also played an important role in the reemergence of whooping cough, despite being attributed to immunity loss^23^.

### Importance of Vaccine Hesitancy in the Brazilian Context: Challenges and Perspectives

Anti-vaccine movements in Brazil are also old. The most well-known manifestation was the *Revolta da Vacina* (Vaccine Revolt) in 1904, with the law of compulsory smallpox vaccination. However, the critical framework of the disease that devastated the country caused the population to quickly seek the vaccine^25^.

Since the 1990s, vaccination coverage in Brazil is high, which reflects the good acceptance by part of the population. The improvement of PNI and the advancement of research, development, and production of immunobiological agents in the country contributed to this success. Certainly, national vaccination campaigns and days favored this adherence, since they expressed public mobilization. Also, communication and media of vaccination actions improved, with simpler language, exploring the ethnic diversity and calling-up national heroes^25^. As direct consequence of the PNI success, vaccine-preventable diseases were controlled^2^.

**Figure 1:**
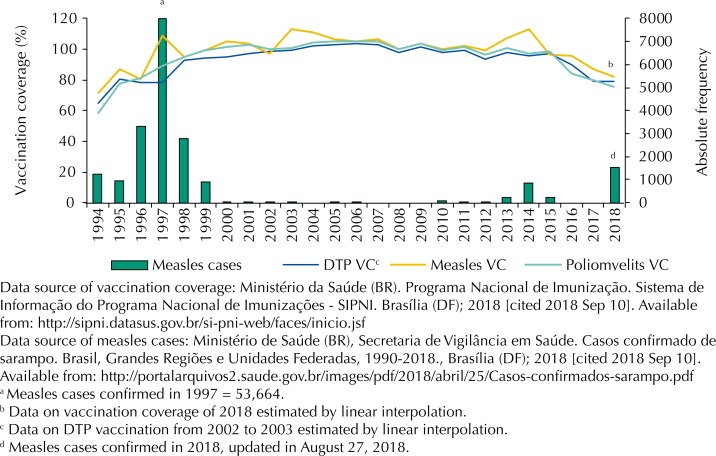
Historical series of vaccination coverage (VC) of diphtheria-tetanus-pertussis (DTP VC), measles (measles VC) and poliomyelitis (Polio VC) and measles cases confirmed from 1994 to 2018, Brazil.

However, this scenario is changing rapidly. Between 2013 and 2015, 1,310 cases of measles were recorded in the states of Ceará and Pernambuco. In 2018, these epidemics reemerged in the states of Roraima and Amazonas with over 1,500 confirmed cases in the first eight months of such year^e^. This statistic is a warning about the impact of the drop in vaccination coverage in general (Figure 1).

**Figure 2:**
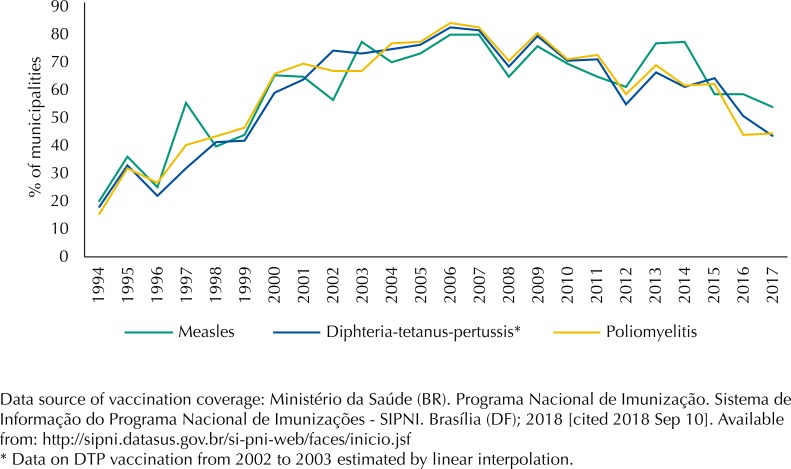
Brazilian municipalities that achieved vaccination coverage (VC) ≥ 95% for child vaccines diphtheria-tetanus-pertussis (DTP) (3rd dose), measles (1st dose) and poliomyelitis (3rd dose), from 1994 to 2017.

Despite high national coverage, homogeneity is still a challenge^5^. Figure 2 shows the proportion of municipalities with coverage of DTP, measles and poliomyelitis vaccines ≥ 95%. One can note the high homogeneity in the first decade of the 2000s and a drop from 2014 on.

**Figure 3:**
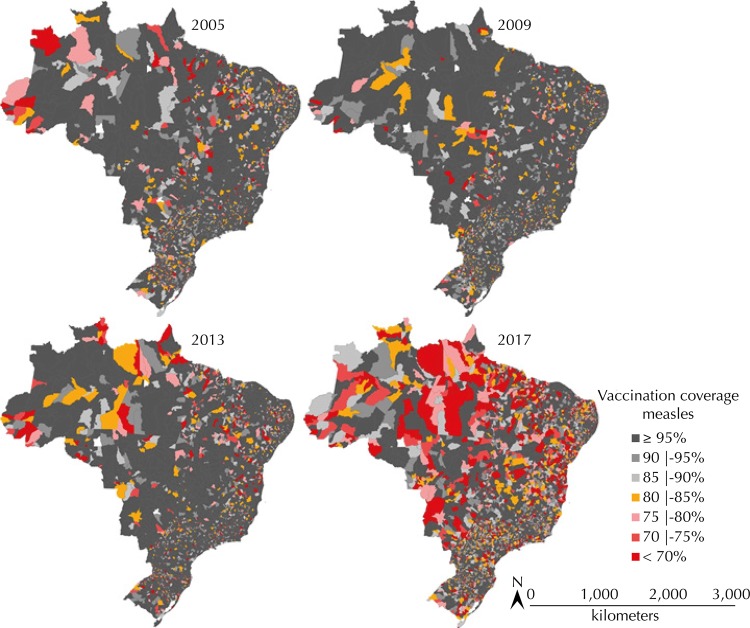
Coverage of measles vaccine (1st dose) in 2005, 2009, 2013 and 2017, according to municipality, Brazil.

Figure 3 presents maps of measles vaccination coverage. Besides the obvious heterogeneity among municipalities, we notice a discreet improvement of coverage between 2005 and 2009 and an important drop between 2013 and 2017. It is worth indicating some reservations about these data, as fluctuations resulting from the territorial size of the municipality and from its population. However, the figure illustrates the importance of continuous surveillance of vaccination coverage and of the quality of vaccination records.

Few Brazilian studies investigated the voluntary refusal or delay to having vaccines available in SUS. Most studies did not name what we currently call vaccine hesitancy. Qualitative studies distinguished groups of parents that vaccinated their children (vaccinators), selective vaccinators and non-vaccinators. These studies show that the vaccination decision is influenced by sociocultural aspects, both unique from the Brazilian context and from the contemporary society^7^ ^,^ ^8^ ^,^ ^26^.

Vaccinator parents state vaccination as an act of duty and responsibility and do it without questioning, being influenced by family tradition and social norm^7^ ^,^ ^8^ ^,^ ^26^. Selective parents experienced different situations that put them in doubt about the decision of vaccinating their children or delaying it, characterizing the singling out of child vaccination, i.e. making it particular, before the extensive vaccination calendar of PNI^7^ ^,^ ^8^ ^,^ ^26^. On the other hand, among non-vaccinators, a more natural vision predominated, i.e. of less medical and hospital intervention in health processes, as well as the autonomy of parental decisions before the regulations on child care. Among the justifications for not vaccinating, there outstood: low perception of disease risk, since they are already under control or mild; fear of adverse events following immunization; questionings about its effectiveness and formulation and about the financial interest of the pharmaceutical industry; option for other forms of health protection^7^ ^,^ ^8^ ^,^ ^26^.

In a study in São Paulo, pity of the child receiving the injections was associated with the delay of measles vaccine for at least 20 days. The authors recommended having two or more communication channels and showed that the vaccine card is a tool that assists vaccination at the preconized age^27^. A review study on adverse events of vaccines provided by SUS showed that the risks associated with vaccines do not justify its disruption and that the risk associated with non-vaccination is growing. The authors also alerted the lack of information and emphasized the role of health professionals^9^. The low proportion of individuals that know the human papillomavirus and its vaccine reinforced this demand for educational interventions^28^. There is evidence of vaccine hesitancy also among health professionals with low coverage of the full schedule of hepatitis B vaccine^29^, which indicates that the importance of vaccination must also be reinforced in the own training and continuing education of these professionals.

The media also has a crucial role in the search for vaccines. The 2007–2008 yellow fever outbreak was portrayed by the press coverage as an epidemic out of control, with no explanation of the sylvatic form of the disease and emphasizing the vaccine as the only salvation. The result was the indiscriminate pursuit of the population for the vaccine, even by people for whom vaccination was contraindicated^30^. From 2017 to 2018, similar problems were experienced, with an exaggerated demand for the vaccine; however, the introduction of fractional-dose vaccine and dispersal of incorrect news made the queues disappear.

## FINAL CONSIDERATIONS

The drop in vaccination coverage instigated Brazilian public administrators and researchers to seek possible explanations. Vaccine hesitancy must be better understood in the Brazilian context. This phenomenon is nothing new in European or North-American countries, and even in Brazil, it has been studied, even if under another name^7^ ^,^ ^8^ ^,^ ^10^ ^,^ ^13^ ^,^ ^14^ ^,^ ^26^.

The poles of total acceptance or total refusal comprise relatively smaller groups than the hesitant ones, which also are distributed into heterogeneous groups. Generally, the interpretation of the vaccine risk is not based on rational evaluation of the evidence, but rather on the sense of uncertainties and ambiguities that remain even in the face of empirical evidence^11^. Thus, several studies emphasize the importance of communication and the link of population with vaccination actions.

Anti-vaccine movements, although ancient, have been gaining strength throughout the world, with a more visible start in high-income countries. However, certainly, the impact of this negative feeling regarding vaccines will be more important in middle- and low-income countries, as these movements are strengthened^14^. Therefore, it is imperative that Brazilian public administrators, researchers and the population mobilize themselves to protect our successful immunization program.

## Footnotes

a Ministério da Saúde (BR). Programa Nacional de Imunização. Sistema de Informação do Programa Nacional de Imunizações - SIPNI. Brasília (DF); 2018 [cited 2018 Sep 10]. Available from: http://sipni.datasus.gov.br/si-pni-web/faces/inicio.jsf

b Ministério da Saúde (BR), Departamento de Informática do Sistema Único de Saúde (DATASUS). Informações de saúde (TABNET). Brasília (DF): 2018 [cited 2018 Sep 10]. Available from: http://www2.datasus.gov.br/DATASUS/index.php/

c Ministério da Saúde (BR). Sarampo: situação epidemiológica. *Inf Epidemiol*. 2018 [cited 2018 Sep 10];(20). Available from: http://portalms.saude.gov.br/saude-de-a-z/sarampo-situacao-epidemiologica

d World Health Organization, Regional Office for Europe. Vaccine-preventable Diseases and Immunization Programme. *WHO EpiData.* 2018 [cited 2018 Sep 10];(7). Available from: http://www.euro.who.int/__data/assets/pdf_file/0008/378602/epi-data-jul2017-jun2018-eng.pdf?ua=1

e Ministério da Saúde (BR). Sarampo: situação epidemiológica. *Inf Epidemiol*. 2018 [cited 2018 Sep 10];(20). Available from: http://portalms.saude.gov.br/saude-de-a-z/sarampo-situacao-epidemiologica
